# Impairment of systemic DHA synthesis affects macrophage plasticity and polarization: implications for DHA supplementation during inflammation

**DOI:** 10.1007/s00018-017-2498-9

**Published:** 2017-03-15

**Authors:** Emanuela Talamonti, Anna M. Pauter, Abolfazl Asadi, Alexander W. Fischer, Valerio Chiurchiù, Anders Jacobsson

**Affiliations:** 1 Department of Molecular Biosciences, The Wenner-Gren Institute, Stockholm University, SE10691 Stockholm, Sweden; 2 European Center for Brain Research, Laboratory of Neurochemistry of Lipids, IRCCS Santa Lucia Foundation, Rome, Italy; 3 Department of Biochemistry and Molecular Cell Biology, University Medical Center Hamburg-Eppendorf, Hamburg, Germany; 4 Department of Medicine, Campus Bio-Medico University of Rome, Rome, Italy

**Keywords:** Omega-3, Macrophages, Inflammation, Lipid metabolism

## Abstract

Docosahexaenoic acid (DHA) is an omega-3 fatty acid obtained from the diet or synthesized from alpha-linolenic acid through the action of fatty acid elongases (ELOVL) and desaturases. DHA plays important roles in the central nervous system as well as in peripheral organs and is the precursor of several molecules that regulate resolution of inflammation. In the present study, we questioned whether impaired synthesis of DHA affected macrophage plasticity and polarization both in vitro and in vivo models. For this we investigated the activation status and inflammatory response of bone marrow-derived M1 and M2 macrophages obtained from mice deficient of Elovl2 (Elovl2^−/−^), a key enzyme for DHA synthesis in mammals. Although both wild type and Elovl2^−/−^ mice were able to generate efficient M1 and M2 macrophages, M1 cells derived from Elovl2^−/−^ mice showed an increased expression of key markers (iNOS, CD86 and MARCO) and cytokines (IL-6, IL-12 and IL-23). However, M2 macrophages exhibited upregulated M1-like markers like CD80, CD86 and IL-6, concomitantly with a downregulation of their signature marker CD206. These effects were counteracted in cells obtained from DHA-supplemented animals. Finally, white adipose tissue of Elovl2^−/−^ mice presented an M1-like pro-inflammatory phenotype. Hence, impairment of systemic DHA synthesis delineates an alteration of M1/M2 macrophages both in vitro and in vivo, with M1 being hyperactive and more pro-inflammatory while M2 less protective, supporting the view that DHA has a key role in controlling the balance between pro- and anti-inflammatory processes.

## Introduction

Docosahexaenoic acid (DHA) is an ω-3 fatty acid prerequisite for normal growth, development, and function in mammals. It cannot be synthesized de novo and, as such, has to be derived from diet or synthesized from dietary α-linolenic acid (ALA) through a series of elongation and desaturation steps performed by distinct enzymes residing in the endoplasmic reticulum [1]. Chain elongation is controlled by the elongation of very-long-chain fatty acid (ELOVLs) enzymes. ELOVL2, involved in the elongation of C22 PUFA, is significantly expressed in liver, testis, uterus, placenta, mammary gland, retina, adipose tissue, and certain areas of the brain, all of which are tissues that are documented as being rich in DHA [2, 3]. Accordingly, Elovl2-ablation results in a dramatic reduction of systemic DHA levels in mice [4, 5]. DHA is a key component of all cell membranes and plays important functions in central nervous system and in peripheral organs. It has been shown that DHA, although is not actively synthesized by immune cells and tissues, is capable of reducing several inflammatory mechanisms that can be of importance in the pathology of metabolic diseases such as cardiovascular disease, diabetes and obesity [6], through mediating processes such as reducing adipocyte differentiation, decreasing adipocyte apoptosis, improving lipolysis as well as insulin metabolism and decreasing production of pro-inflammatory cytokines [7]. In fact, DHA increases both the number of macrophages containing ingested particles and the number of phagocytized particles in adipose tissue, and also reduces macrophage reactive oxygen species production [8, 9]. Macrophages are key players in various host protective processes ranging from protection against pathogen infections to clearance of cellular debris and tissue repair and regeneration, with different macrophage subsets being associated with the propagation or resolution of inflammation [10, 11]. The two extremes in the spectrum of macrophage function are represented by the classically activated (M1) and the alternatively activated M2 macrophages. In general, M1 macrophages are efficient producers of proinflammatory cytokines, whereas macrophages displaying the M2 phenotype are anti-inflammatory/pro-resolving [12]. Furthermore, DHA is also the precursor of a brand-new discovered class of molecules, commonly addressed to as “pro-resolving lipid mediators” (SPMs) that include resolvins (E- and D-series), protectins and maresins, which are involved in the phases of resolution of inflammation [13]. The production of SPMs is very important in acute inflammatory response and requires the involvement of different cells including macrophages [14]. On this basis, the aim of the present study was to investigate whether impaired synthesis of DHA in Elovl2^−/−^ mice affected macrophage plasticity and polarization both in vitro and in vivo models. To address this question we performed a wide screening of the main M1 and M2 markers by PCR, flow cytometry and ELISA in bone marrow-derived macrophages and in white adipose tissue obtained from mice deficient for the Elovl2 enzyme. Our results have showed for the first time that impairment of systemic DHA synthesis delineates an immunophenotypic alteration of M1/M2 macrophages, with M1 being more pro-inflammatory and M2 less protective, supporting the view that DHA has a key role in controlling the balance between pro- and anti-inflammatory process.

## Materials and methods

### Animals

Elovl2^−/−^ mice were generated as described previously [4, 15] from Elovl2-ablated embryonic stem cells (derived from 129/Sv agouti mice) injected into C57BL/6J blastocysts to generate offspring heterozygous for the mutations and then backcrossed into the 129S2/Sv strain for five generations. All animals were housed at room temperature and maintained on a 12-h light/dark cycle. 18- to 24-week-old male or female mice were fed standard chow DHA-free diet (10% kcal fat, D12450H, Research Diets, New Brunswick, NJ, USA) or DHA-enriched (10% kcal fat, 1% DHA, D13021002, Research Diets, New Brunswick, NJ, USA) for 3 months, according to the experimental groups. Dietary fatty acid composition and other nutrients are shown in Tables 1 and 2. All animals were fed ad libitum and had free access to water. At the end of the study, animals were euthanized with CO_2_ and killed by cervical dislocation. Studies were carried out with ethical permission from the Animal Ethics Committee of the North Stockholm region, Sweden.

**Table 1:** Diet composition. Diet composition expressed in gram of mass for each ingredient, of control diet (10% kcal fat, D12450H, Research Diets, New Brunswick, NJ, USA) and DHA-enriched diet (10% kcal fat, 1% DHA, D13023002, Research Diets, New Brunswick, NJ, USA) formulated by Research Diets, New Brunswick, NJ, USA

Fatty acid	Control diet (10% kcal fat) D12450H	DHA-enriched diet (10% kcal fat, 1% DHA) D13021002
C12:0	0.09	0.07
C14:0	0.80	0.48
C15:0	0.05	0.05
C16:0	16.83	12.48
C16:1n-9	0.17	0.09
C16:1n-7	0.86	0.51
C18:0	8.43	5.35
C18:1n-9	27.87	21.35
C18:1n-7	1.66	1.29
C18:2n-6*	37.43	35.64
C18:3n-6	0.16	0.19
C18:3n-3**	4.24	4.46
C20:0	0.30	0.23
C18:4n-3	0.05	0
C20:1n-9	0.37	0.25
C20:2n-6	0.31	0.19
C20:4n-6	0.11	0.24
C22:0	0.24	0.20
C20:4n-3	0	0.03
C20:5n-3	0	0.71
C22:3n-3	0	0.08
C22:4n-6	0	1.04
C22:5n-3	0	0.57
C22:6n-3***	0	14.53
	99.97	100.03

*LA (linoleic acid)

**ALA (α-linolenic acid)

***DHA (docosahexaenoic acid)

**Table 2:** Diet fatty acid composition. Fatty acids expressed as % of total fatty acids of control diet (10% kcal fat, D12450H, Research Diets, New Brunswick, NJ, USA) and DHA-enriched diet (10% kcal fat, 1% DHA, D13023002, Research Diets, New Brunswick, NJ, USA)

Ingredient	Control diet (10% kcal fat) D12450H	DHA-enriched diet (10% kcal fat, 1% DHA)D13021002
Casein	200	200
L-Cystine	3	3
Corn starch	452.2	452.2
Maltodextrin 10	75	75
Sucrose	172.8	172.8
Cellilose, BW200	50	50
Soybean oil	25	25
Lard	10	10
Mineral mix S10026	10	10
DiCalcium phosphate	13	13
Calcium carbonate	5.5	5.5
Potassium citrate,1H20	16.5	16.5
Vitamin mix V1001	10	10
Choline bitartrate	2	2
FD&C yellow dya #5	0.04	0
FD&C red dya #40	0.01	0.025
FD&C blue dya #1	0	0.025
Total (g)	1055.05	1055.05

### Isolation and culture of bone marrow macrophages

Bone marrow-derived macrophages (BMM) were prepared by culturing bone marrow cells obtained from the femur and tibia of mice in complete RPMI 1640 medium with 5% FBS and 40 ng/ml macrophage colony-stimulating factor (M-CSF) (both purchased from Miltenyi Biotec, CA, USA) for 6 days, and medium was replaced every second day. Subtype-specific polarization and activation of M1 and M2 macrophages was obtained by adding interferon IFN-γ (10 ng/ml) plus LPS (1 μg/ml) or IL-4 (20 ng/ml), respectively, in the culture medium for 2 additional days, respectively.

### Isolation of adipose tissue

Visceral white adipose tissue (WAT) was obtained from epididymal deposit of both WT and Elovl2^−/−^ mice. The tissue was minced and digested with collagenase (250 U/ml in PBS, 2% bovine serum albumin (BSA), pH 7.4, v/v) for 30 min at 37 °C and the cell suspension was filtered through a 250-mm filter. Cells from the stroma-vascular fraction (SVF) were obtained after centrifugation at 300×*g* (room temperature) and treatment with erythrocyte lysis buffer (Sigma Aldrich) for 5 min. Finally, matrix fragments were removed using successive filtrations through 70 and 40-mm nylon meshes. Cells from SVF were then analyzed for cell surface markers by flow cytometry.

### Flow cytometry

Differentiated macrophages were stained for surface markers using the following antibodies: CD45-FITC (1:100), F4/80-APC (1:100), CD86-APC-Cy7 (1:50), CD80-BV421 (1:50) and CD206 (1:100). After 30 min of incubation the cells were washed in PBS and surface receptor expression was analyzed by FACSVerse flow cytometer (BD Biosciences), as reported [16]. Data were analyzed with the FlowJo Software (TreeStar, Ashland, OR, USA).

### ELISA

Macrophages supernatants were collected and stored at −20 °C until usage. Levels of cytokines IL-6, IL-12p70 and IL-23 and chemokine CCL17 were measured by specific ELISA Kits (eBioscience and R&D system), according to the manufacture’s instructions, as reported [17]. The optical density was determined using a micro-plate reader (Molecular Devices Corp, Sunnyvale, CA, USA) set at 450 nm.

### qRT-PCR

RNA was isolated with TRI Reagent (Sigma Aldrich), and total RNA was isolated following the manufacturer’s procedure. For real-time PCR, 500 ng total RNA was reverse transcribed using random hexamer primers, deoxynucleoside triphosphates, MultiScribe reverse transcriptase, and RNase inhibitor (Applied Biosystems, Foster City, CA). The following program was used for the quantitative RT-PCR: 25 °C for 10 min, 42 °C for 50 min, 85 °C for 5 min, then after addition of 0.1 unit/ml of RNase H, the product was incubated at 37 °C for 20 min. Gene-specific primers were premixed with 11 μl of SYBR Green JumpStart Taq Ready Mix (Sigma–Aldrich, S4438) to a final concentration of 0.3 μM. cDNA was diluted 1:10, and aliquots of 2 μl per reaction were run in duplicate. Thermal cycling conditions were 2 min at 50 °C, 10 min at 95 °C, and 40 cycles of 15 s at 95 °C and 1 min at 60 °C, followed by melting curve analysis on a Bio-Rad CFX Connect Real-Time system. Actin and Transcription Factor IIB (*TFIIB*) were used as housekeeping genes for quantity normalization. Primers used were Arginase 1 (*Arg-1*), *STAT6, MARCO*, inducible nitric oxide synthase (*iNOS*), interleukin 1*β* (*IL-1β*), lipoxygenases 5-LOX, 12-LOX, 15-LOX, cyclooxygenases *COX-1* and *COX-2*, adipocyte Protein 2 (aP2), adiponectin, leptin and TNF-α. For primers sequences, see Table 3.

**Table 3:** Murine primers used for the quantitative RT-PCR

	Forward	Reverse
*Arg-1*	GGGAGGCCTATCTTACAGAGAA	GAGTTGGGTTCACTTCCATGAT
*STAT6*	CCTTGGAGAACAGCATTCCTGG	GCACTTCTCCTCGGTGACAGAC
*MARCO*	CCAAGCTATGTTCCCTGTGAT	GACTGCCATGCAGAAGGT
*iNOS*	TCTCCCTTTCCTCCCTTCTT	CTTCAGTCAGGAGGTTGAGTTT
*IL-1β*	TGCCACCTTTTGACAGTGATG	AAGGTCCACGGGAAAGACAC
*LOX-5*	CGGGAACAGCTTATCTGCGA	GTCAGATCCTGGACAGCCCTC
*LOX-12*	CACACATGGTGAGGAAATGG	GATCACTGAAGTGGGGCTGT
*COX-1*	GGTAGTTGTCGAGGCCAAAG	GTCCTGCTCGGAGATGGT
*COX-2*	CCGTAGCTGGTTGGAGTGG	CTGAAGGGTCCGGGAGAT
Adiponectin	GAGATGCAGGTCTTCTTGGTC	CCTGTCATTCCCAACATCTCC
AP2	CGCAGACGACAGGAAGGT	TTCCATCCCACTTCTGCAC
TNFα	CCACATCTCCCTCCAGAAA	CTTCTGCCAGTTCCACGTC
Leptin	GTGGTGGCTGGTGTCAGAT	TTGATGAGGTGACCCAAGGT
Actin	AGTCCCTGCCCTTTGTACACA	CGATCCGAGGGCCTCACTA
TFIIB	TGGAGATTTGTCCACCATGA	GAATTGCCAAACTCATCAAAACT

### Immunofluorescence

For immunohistochemical staining of M1 macrophages in epididymal adipose tissue, the tissues were harvested and fixed in alcoholic formaldehyde (4% formaldehyde in 95% ethanol). After dehydrating and embedding in paraffin, 5 µm sections were cut on a Leica microtome (RM2255, Leica Microsystems) and mounted on SuperFrost microscopy slides (Thermo Fisher). Sections were deparaffinized, rehydrated and boiled for 10 min in target retrieval solution (DAKO) to unmask antigens. Afterwards, autofluorescence was blocked by incubation in 0.3% Sudan black in 75% ethanol for 10 min. The slides were rinsed with PBS and then blocked for 1 h at RT in Blocking Buffer (Roth). Anti-iNOS antibody (#129372, Abcam, 1:200) and anti-Perilipin antibody (#9349, Cell signaling, 1:1000) were diluted in Blocking Buffer (BB) and slides were incubated in primary antibodies over night in a humid chamber at 4 °C. After 3 times 10-min washing, slides were incubated for 1 h at RT in secondary antibodies (Jackson Immunoresearch, Cy2-anti rabbit and Cy3 anti-mouse, both 1:500 in BB). After additional 3 times washing in PBS, slides were mounted using Prolong Gold Antifade Mountant (Thermo Fisher). Microscopy was performed on a Nikon A1 confocal microscope (Nikon) using a Plan Fluor 40× Oil objective. Image analysis was performed using NIS Elements Advanced Research software (Nikon).

### Western Blot

Tissue samples were lysed in RIPA buffer supplemented with complete mini protease inhibitor cocktail (Roche) and 50 mM Na_3_VO_4_ using a TissueLyzer (Qiagen). 20 µg protein was separated on 10% Tris–glycine gels. Transfer to a nitrocellulose membrane (GE) was performed in a wet blotting system for 2 h at 400 mA. Membranes were then blocked for 1 h in 5% milk in TBS-Tween (TBST) and after rinsing with TBST, membranes were incubated in the respective primary antibody overnight at 4 °C. Primary antibodies and dilutions were: CD36 (Novus Biologicals, 1:1000); LRP1 (Abgent, 1:25,000); LPL (Kind gift from Stefan K. Nilsson, 1:1000), SCD1 (Santa Cruz, 1:250), LPL (Cell Signaling, 1:1000). All primary antibodies were diluted in 5% BSA in TBS-T. After incubation, the membranes were washed 3 times 10 min in TBST and incubated for 1 h with HRP-coupled secondary antibodies (all from Jackson, all 1:5000 in 5% milk in TBS-T). After 3 additional washes in TBST, detection was performed using Amersham ECL Prime Western Blotting Detection Reagent (GE) and Amersham Hyperfilm (GE) autoradiography films. The films were developed using Kodak Developer and Fixer.

### Statistical analysis

Data were expressed as mean ± SD and were analyzed by means of the Prism 4 software (GraphPad Software, San Diego, CA). Differences between two groups were analyzed using Student’s *t* test. Multiple comparisons will be performed by ANOVA with a Bonferroni post hoc test. A *p* value < 0.05 was considered significant. FACS analysis was performed using the Flowjo analysis program (Treestar, Ashland, OR).

## Results

### DHA deficiency increases the pro-inflammatory phenotype of M1 macrophages

**Fig. 1:**
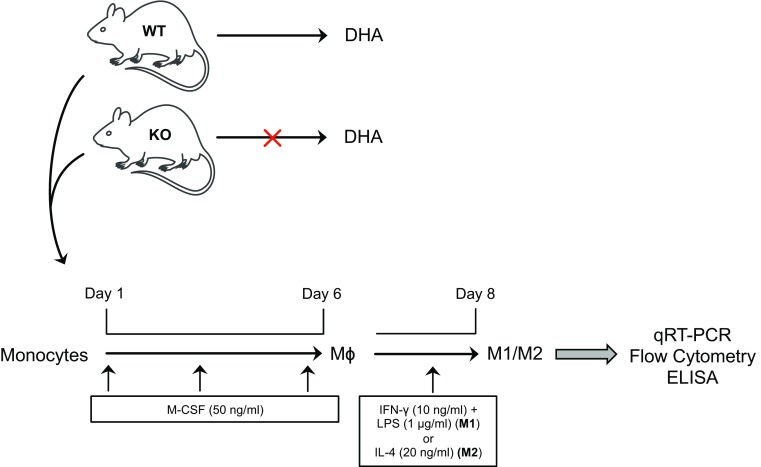
Schematic representation of M1 and M2 macrophage polarization and activation in Elovl2^−/−^ and WT mice fed without DHA

**Fig. 2:**
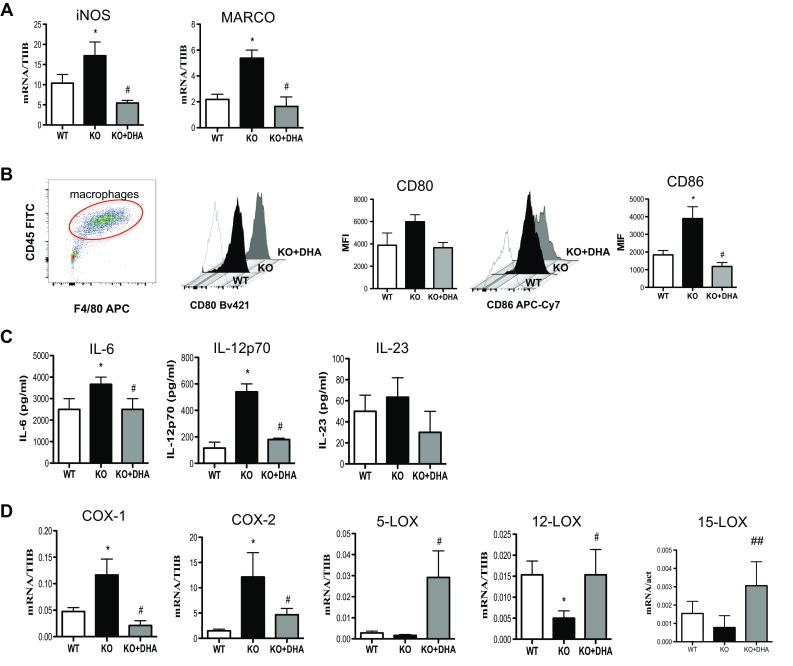
Analysis of M1 markers in macrophages obtained from Elovl2^−/−^ and WT mice fed with diet enriched or not in DHA. Monocytes obtained from bone marrow of Elovl2^−/−^ (KO) and WT mice fed with diet enriched or not in DHA were differentiated into M1 macrophages using M-CSF (50 ng/ml) and IFN-γ (10 ng/ml) plus LPS (1 μg/ml). **a** mRNA analysis of iNOS and MARCO. Data are shown as mean (±sem) of ten independent experiments, each in duplicate. **b** CD80 and CD86 amount analyzed by flow cytometry upon staining cells at cell surface. Data are reported as mean fluorescence intensity (MFI), and are representative of eight independent experiments ± sem **c** ELISA of IL-6, IL-12p70 and IL-23 levels in supernatants of M1 macrophages. Data are reported as pg/ml, and are representative of five independent experiments ± sem, each in duplicate. **d** mRNA analysis of cyclooxygenases (COX-1 and COX-2) and lipoxygenases (5-LOX, 12-LOX, 15-LOX). *Denotes *p* < 0.05 versus WT; **Denotes *p* < 0.01 versus WT; #Denotes *p* < 0.05 versus KO; ^##^denotes *p* < 0.01 versus KO

As already shown by our previous studies where total serum DHA levels were drastically reduced in Elovl2^−/−^ mice [4, 5] and given the important role of DHA in inflammatory responses, we examined whether the impairment of systemic DHA synthesis could modulate the immunophenotype of bone marrow-derived M1 and M2 macrophages obtained from WT and Elovl2^−/−^ mice (Fig. 1). According to the new guidelines recently given by Murray et al. [12] on how to obtain M1 and M2 macrophages and which markers to follow, we polarized macrophages from murine bone marrow and investigated a pool of markers to describe M1 and M2 activation accordingly (Fig. 1). As shown in Fig. 2, M1 macrophages obtained from Elovl2^−/−^ mice showed a significant increased mRNA expression of both signature markers iNOS (~45%) and MARCO (~40%) (Fig. 2a), and an upregulation of activation markers CD80 (25%) and CD86 (50%) (Fig. 2b) compared to WT M1 macrophages. Furthermore, M1 of Elovl2^−/−^ mice were also functionally altered inasmuch as they released higher levels of the pro-inflammatory cytokines IL-6 (3667.0 ± 333.0 versus 2500.0 ± 500.0), IL-12 (540.0 ± 60.0 versus 115.0 ± 45.0) and IL-23 (63.3 ± 18.6 versus 50.0 ± 15.3) compared to WT M1 macrophages (Fig. 2c). Interestingly, such upregulation of M1 markers was counteracted following DHA supplementation in the diet of Elovl2^−/−^ mice (Fig. 2a–c) whereas DHA supplementation in WT animals did not have any significant effect (data not shown), suggesting that in DHA-deficient animals M1 macrophages are hyperactive and more pro-inflammatory. We next sought to analyze the mRNA expression of the main enzymes involved in AA, EPA and DHA metabolism, i.e. cyclooxygenases and lipoxygenases. As expected, classically activated M1 macrophages from Elovl2^−/−^ mice showed a 2-fold induction of both constitutive COX-1 and inducible COX-2 expression compared to WT mice (Fig. 2d). However, when DHA was supplemented in the diet, only the expression of COX-2 was reverted. For lipoxygenases, which are required for SPMs production from EPA and DHA, our results showed a reduced expression of 5-LOX, 12-LOX and 15-LOX, which was significant only for 12-LOX, in M1 macrophages of Elovl2^−/−^ mice compared to WT mice. This was significantly reverted upon DHA supplementation (Fig. 2e). Although the difference in basal 5-LOX expression between WT and Elovl2^−/−^ cells was not significant, supplementation of Elovl2^−/−^ mice with DHA resulted in a determined 10-fold increase of 5-LOX.

### DHA deficiency attenuates the anti-inflammatory phenotype of M2 macrophages

**Fig. 3:**
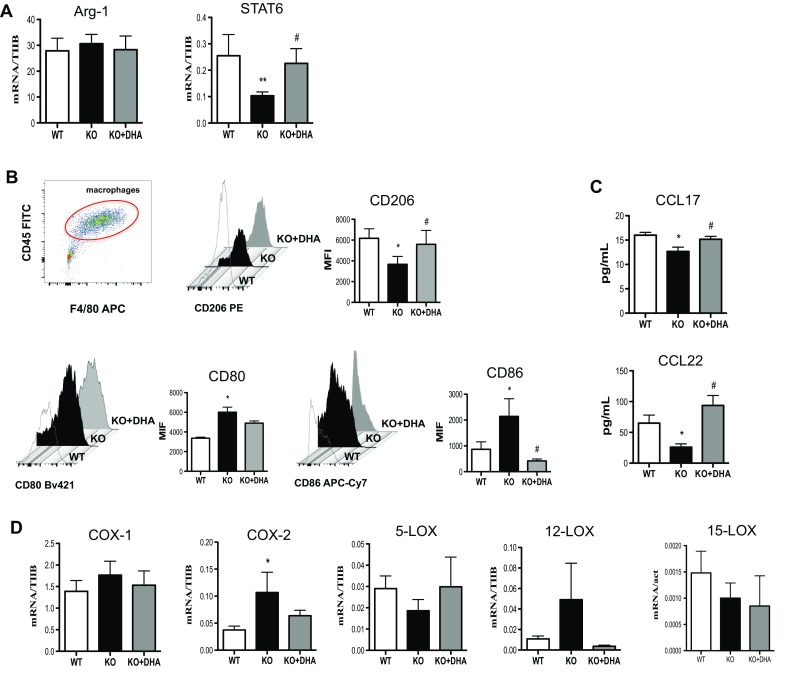
Analysis of M2 markers in macrophages obtained from Elovl2^−/−^ and WT mice fed with diet enriched or not in DHA. Monocytes obtained from bone marrow of Elovl2^−/−^ (KO) and WT mice fed with diet enriched or not in DHA and differentiated into M2 using M-CSF (50 ng/ml) and IL-4 (20 ng/ml). **a** mRNA analysis of Arg-1 and STAT6 by RT-PCR. Data are shown as mean (±sem) of ten independent experiments, each in duplicate. **b** CD206, CD80 and CD86 amount by flow cytometry upon staining cells at cell surface. Data are reported as mean fluorescence intensity (MFI), and are representative of eight independent experiments ± sem **c** ELISA of CCL-17 and CCL-22 levels in supernatants of M2 macrophages. Data are reported as pg/ml, and are representative of five independent experiments ± sem, each in duplicate. **d** mRNA analysis of cyclooxygenases (COX-1 and COX-2) and lipoxygenases (5-LOX, 12-LOX, 15-LOX). *Denotes *p* < 0.05 versus WT; **denotes *p* < 0.01 versus WT. #Denotes *p* < 0.05 versus KO

We also questioned whether the immunophenotypical profile of M2 macrophages following impaired DHA synthesis was affected. As shown in Fig. 3, M2 macrophages obtained from Elovl2^−/−^ mice showed a significant downregulation in the expression of STAT6 transcription factor (~60%) (Fig. 3a) and surface protein CD206 (~50%) (Fig. 3b) compared to WT mice, while no variation of Arg-1 mRNA expression was observed in all experimental conditions (Fig. 3a). Intriguingly, the Elovl2^−/−^ M2 macrophages exhibited a significant upregulation of M1-like markers CD80 and CD86 (Fig. 3b), suggesting an M2-to-M1 switch during impaired DHA synthesis. Furthermore, in Elovl2^−/−^ mice M2 macrophages released lower levels of both Th2-inducing chemokines CCL17 and CCL22 (Fig. 3c) compared to WT mice. This M2 immunophenotype in Elovl2^−/−^ mice was significantly reverted when these mice were supplemented with DHA in their diet (Fig. 3a–c), suggesting that the presence of DHA might be crucial for the M2-mediated anti-inflammatory responses.

Additionally, we observed a slight upregulation of COX-1 expression (~20%) and a significant upregulation of COX2 (~40%) in M2 macrophages from Elovl2^−/−^ mice compared to WT mice, which was counteracted once again by DHA supplementation (Fig. 3d). For lipoxygenases, 5-LOX and 15-LOX mRNA levels were downregulated, although not significantly, in M2 cells of Elovl2^−/−^ mice and DHA supplementation was only able to revert 5-LOX levels but not 15-LOX (Fig. 3d). Interestingly, M2 cells of Elovl2^−/−^ mice upregulated LOX-12 expression compared to WT mice, which was reversed by DHA supplementation (Fig. 3d). These findings suggest that impairment of DHA synthesis might, as for M1 cells, also affect the ability of M2 macrophages to modulate the production of COX- and LOX-derived pro-inflammatory or resolution mediators.

### M1/M2 immunophenotype is altered in vivo within white adipose tissue and affects its properties

**Fig. 4:**
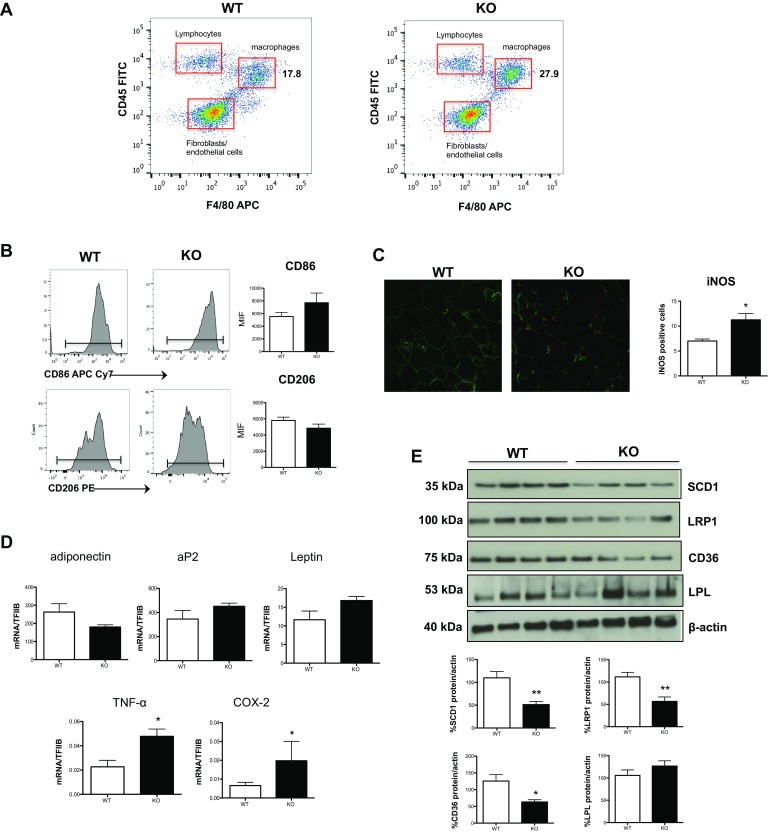
Analysis of inflammatory and adipogenic state of white adipose tissue. SVF was isolated from WAT of both WT and Elovl2^−/−^ mice. **a** Gating strategy to analyze the amount of macrophages (F4/80) present in the cell fraction excluding lymphocytes (F4/80-CD45+) and fibroblast and endothelial cells (F4/80-CD45−). **b** CD206 and CD86 amount by flow cytometry upon staining cells at cell surface. Data are reported as mean fluorescence intensity (MFI), and are representative of three independent experiments. **c** Immunofluorescence of iNOS was performed by confocal laser-scanning microscopy, and data are shown as pictures taken with a Plan Fluor 40× Oil objective and as analysis (mean ± SD) of five independent experiments. **d** mRNA analysis of adiponectin, aP2, leptin and pro-inflammatory markers TNF-α and COX-2 in WAT. Data are shown as mean (±SD) of four independent experiments, each in duplicate. **e** Western blot analysis of SCD1, LRP1, CD36 and LPL, with the expected molecular mass of each protein shown on the left-hand side. Data are expressed in comparison with β-actin, and are the mean (±sem) of four different mice. *p < 0.05 versus WT; **denotes *p* < 0.01 versus WT

To obtain in vivo evidence of the M1/M2 imbalance phenotype observed in polarized monocytes obtained from DHA-deficient Elovl2^−/−^ mice, we next investigated the abundance of M1 and M2 macrophages within the stromal vascular fraction (SVF) from white adipose tissue (WAT), whose lipid and inflammatory homeostasis is known to be strictly regulated by these two cell types. At first, we observed that the SVF of Elovl2^−/−^ mice was richer in total macrophages (CD45 + F4/80+) compared to WT mice, while the other cell fractions (i.e. CD45+F4/80-lymphocytes and CD45-F4/80- fibroblasts and endothelial cells) remained constant (Fig. 4a). Furthermore, the immunophenotypical analysis of such increased WAT macrophages revealed an increased expression in M1 markers CD86 (Fig. 4b) and iNOS (Figs. 4c) concomitantly with a slightly reduced expression of M2 marker CD206 in Elovl2^−/−^ compared to WT mice, suggesting that DHA deficiency favours an accumulation of M1 over M2 macrophages within tissues.

Since DHA deficiency-induced accumulation of M1 macrophages in WAT could potentially affect the functions of this tissue, we then evaluated the expression of several genes associated with adipocyte differentiation, lipid storage, obesity and inflammation. Interestingly, WAT from Elovl2^−/−^ mice showed a non-significant reduction in mRNA expression of adiponectin and an increase of adipocyte protein 2 (aP2) and leptin as well as a significant increase in the expression of the inflammatory markers TNF-α and COX-2 (Fig. 4d). Furthermore, to get more information on how lipid metabolism is affected by DHA deficiency, several markers involved in de novo lipogenesis (SCD-1) and in lipid uptake (LRP1, CD36, LPL) were analyzed. In particular, immunoblotting analysis revealed a significant decrease of SCD-1 and of the two scavenger receptors LRP1 and CD36 in Elovl2^−/−^ compared to WT mice, whereas the expression levels of LPL were slightly increased (Fig. 4e) suggesting that DHA deficiency determines alterations in adipose tissue lipid metabolism and inflammation.

## Discussion

**Fig. 5:**
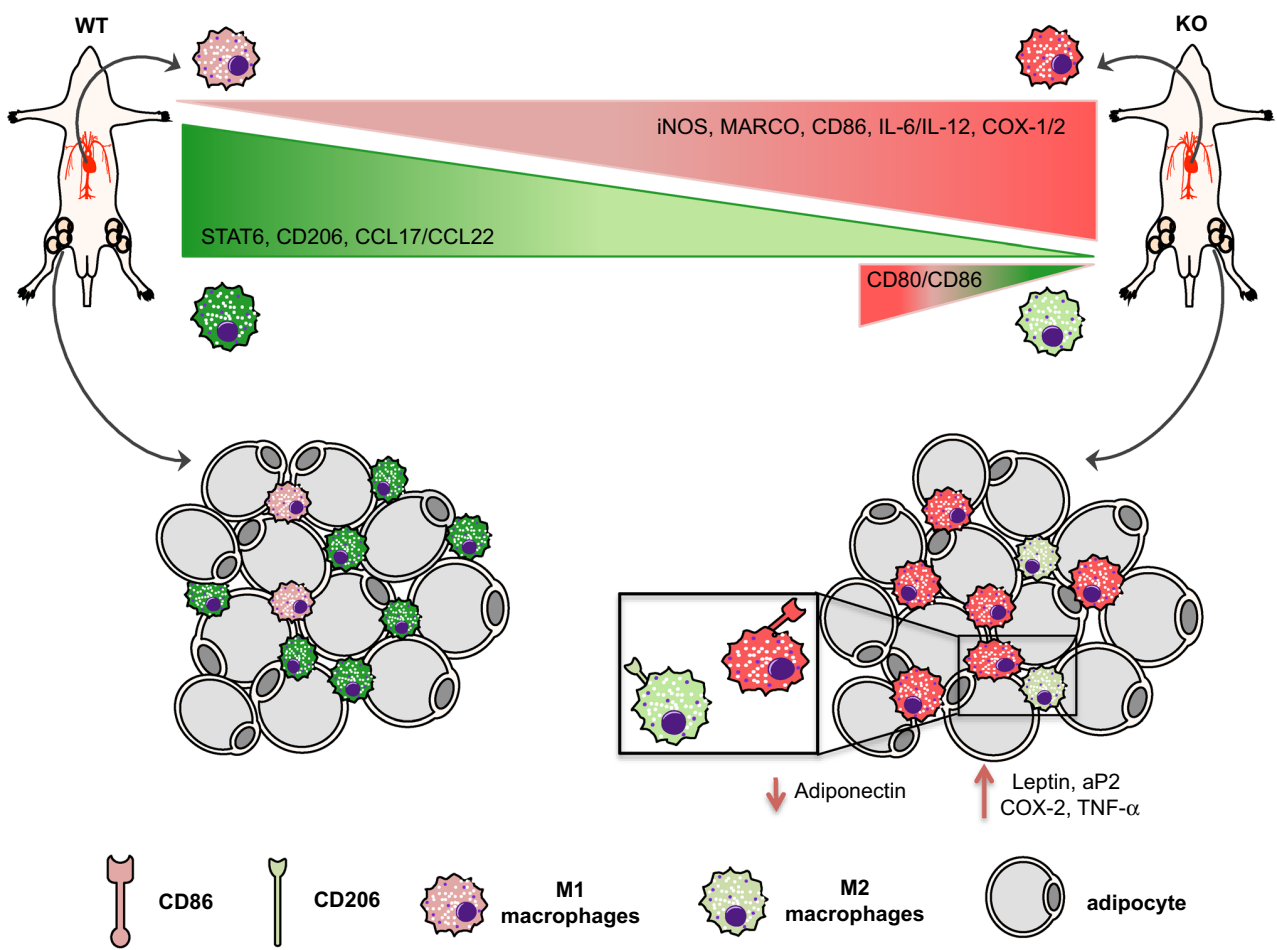
Summary of DHA deficiency-induced influence on macrophage phenotype and adipose tissue inflammation. Impairment of systemic DHA synthesis delineates an immunophenotypic alteration of M1/M2 macrophages both in vitro and in vivo, with M1 being hyperactive and more pro-inflammatory while M2 less protective. This has implications also on adipose tissue metabolism and functions

The beneficial properties of dietary omega-3 polyunsaturated fatty acids (PUFA), in particular of DHA, have been recognized for long and their metabolic dysfunction have been linked to a range of diseases including various inflammatory disorders, cardiovascular diseases, cancer and obesity [18]. Numerous in vitro and in vivo studies suggest that DHA, although not produced by immune cells but obtained from the blood, attenuates inflammatory properties of monocytes/macrophages [19–21], whose role in the immunopathogenesis of many inflammatory and metabolic diseases is constantly expanding [22, 23]. However, the specific role of DHA on the activation status and the immunological responses of macrophages is still scarce. Hence, in this study we interrogated whether endogenously synthesized DHA could affect macrophage plasticity and polarization. To do this, we used mice deficient for Elovl2, a key enzyme involved in the synthesis of DHA. In the present work, we collected evidence for an alteration of the balance between classically activated M1 and alternatively activated M2 macrophages in Elovl2^−/−^ mice. Such alteration affected not only macrophage phenotype but also their ability to produce inflammatory mediators (Fig. 5). Indeed, although both WT and Elovl2^−/−^ mice were able to generate efficient M1 and M2 macrophages, M1 derived from Elovl2^−/−^ mice showed an overall upregulation of several of their signature markers (iNOS, MARCO, CD80 and CD86) and increased expression of pro-inflammatory cytokines (IL-6, IL-12 and IL-23) whereas, M2 macrophages not only showed a reduction of their anti-inflammatory phenotype but also a concomitant switch to an M1-like phenotype. This is particularly important because such markers are strictly associated with the promotion of a pro-inflammatory environment and the subsequent induction of T-cell activation [24] and generation of highly pathogenic T-helper 1 and T-helper 17 cells [25, 26]; all these processes are critically involved in the immunopathogenesis of many chronic inflammatory or autoimmune diseases. On the other hand, the loss of the M2 immunoregulatory and anti-inflammatory phenotype observed in DHA-deficient Elovl2^−/−^ mice could endow them with a reduced ability to recruit T-helper 2 and regulatory T cells, whose activity is crucial to maintain a tolerogenic environment or to efficiently contrast hyperactive and sustained inflammatory processes. These findings were corroborated by our observation that when supplementing the diet of Elovl2^−/−^ mice with DHA, the M1/M2 imbalance was reverted, suggesting that DHA impairment significantly impacts on macrophage plasticity and functions by either enhancing the pro-inflammatory status of M1 macrophages or by reprogramming M2 macrophages destiny from an anti-inflammatory to a pro-inflammatory one. Furthermore, our observed alteration in the expression of lipoxygenases 5-LOX, 12-LOX and 15-LOX in Elovl2^−/−^ mice is of note, since these enzymes are involved also in the conversion of DHA into the newly discovered SPMs resolvins, maresins and protectins, which play an important role in the phases of resolution of inflammation and in the immunomodulation of T cell responses [27, 28]. Indeed, the discovery of a range of bioactive mediators derived from DHA that possess potent anti-inflammatory and pro-resolving properties may be responsible, at least in part, for our observed beneficial effects associated with omega-3 fatty acids. For instance, Resolvin D1 (aDHA-derivedSPM) has been reported to increase both the number of macrophages containing ingested particles and the number of phagocytized particles in adipose tissue, and also reduces macrophage reactive oxygen species production [29].

Since macrophage plasticity and polarization are strongly influenced by the surrounding microenvironment and the amount of omega-3 is significantly altered in several pathological states, the ability of DHA to affect the M1/M2 balance is of great importance and highlights endogenous omega-3 fatty acids synthesis and signaling as a novel endogenous mechanism for affecting macrophage biology and plasticity. This hypothesis was also corroborated by our in vivo findings, where we observed that such imbalance between M1 and M2 macrophages was also present in white adipose tissue. Inflammation originating from the adipose tissue is considered to be one of the main driving forces for the development of insulin resistance and diabetes in obese individuals [30]. Adipose tissue is also an immune organ since it is not only made up by adipocytes and preadipocytes but also is a resident of immune cells including macrophages, T and B cells. Inflamed WAT can indeed express several pro-inflammatory markers including TNF-α and COX-2 as well as present alterations in insulin-sensitizing proteins such as adiponectin [31] or differentiation and lipid-uptake receptors including leptin, aP2, SCD1 and CD36 [32]. For instance, increased leptin secretion by hypertrophic adipocytes also activates T cells to secrete IFN-γ, which causes adipocytes to express the antigen presenting molecule MHC-II, suggesting that adipocytes themselves may participate in immune activation [33]. Furthermore, several studies have shown the immunoregulatory role of DHA and its involvement in reducing adipocyte differentiation, adipocyte apoptosis, improving lipolysis as well as controlling the production of pro- and anti-inflammatory molecules [34].

In conclusion, we have highlighted here the important role of the DHA-generating Elovl2 enzyme in the modulation of the balance between M1/M2 macrophages both in vitro and in vivo, with conceivable and potential implications in affecting adipose tissue inflammation and lipid metabolism. Indeed, our findings suggest that the enzymatic machinery that leads to DHA synthesis is involved, at least in part, in the maintenance of innate immune functions and, on one hand, mechanistically support the notion that diets rich in DHA are beneficial against the onset of macrophage-driven chronic inflammatory diseases and, on the other hand, propose Elovl2 as a pharmacological target for the treatment of DHA deficiency in mammals.

## References

[1] Guillou H, Zadravec D, Martin PG, Jacobsson A (2010). The key roles of elongases and desaturases in mammalian fatty acid metabolism: Insights from transgenic mice. Prog Lipid Res.

[2] Tvrdik P, Westerberg R, Silve S, Asadi A, Jakobsson A, Cannon B, Loison G, Jacobsson A (2000). Role of a new mammalian gene family in the biosynthesis of very long chain fatty acids and sphingolipids. J Cell Biol.

[3] Ohno Y, Suto S, Yamanaka M, Mizutani Y, Mitsutake S, Igarashi Y, Sassa T, Kihara A (2010). ELOVL1 production of C24 acyl-CoAs is linked to C24 sphingolipid synthesis. Proc Natl Acad Sci USA.

[4] Pauter AM, Olsson P, Asadi A, Herslöf B, Csikasz RI, Zadravec D, Jacobsson A (2014). Elovl2 ablation demonstrates that systemic DHA is endogenously produced and is essential for lipid homeostasis in mice. J Lipid Res.

[5] Pauter AM, Trattner S, Gonzalez-Bengtsson A, Talamonti E, Asadi A, Dethlefsen O, Jacobsson A (2017). Both maternal and offspring Elovl2 genotypes determine systemic DHA levels in perinatal mice. J Lipid Res.

[6] Singhal A, Lanigan J, Storry C, Low S, Birbara T, Lucas A, Deanfield J (2013). Docosahexaenoic acid supplementation, vascular function and risk factors for cardiovascular disease: A randomized controlled trial in young adults. J Am Heart Assoc.

[7] Murumalla RK, Gunasekaran MK, Padhan JK, Bencharif K, Gence L, Festy F, Cesari M, Roche R, Hoareau L (2012). Fatty acids do not pay the toll: effect of sfa and pufa on human adipose tissue and mature adipocytes inflammation. Lipids Health Dis.

[8] Titos E, Rius B, Gonzalez-Periz A, Lopez-Vicario C, Moran-Salvador E, Martinez-Clemente M, Arroyo V, Claria J (2011). Resolvin d1 and its precursor docosahexaenoic acid promote resolution of adipose tissue inflammation by eliciting macrophage polarization toward an m2-like phenotype. J Immunol.

[9] Vieira-Potter VJ (2014). Inflammation and macrophage modulation in adipose tissues. Cell Microbiol.

[10] Gordon S, Mantovani A (2011). Diversity and plasticity of mononuclear phagocytes. Eur J Immunol.

[11] Gautier EL, Shay T, Miller J, Greter M, Jakubzick C, Ivanov S, Helft J, Chow A, Elpek KG, Gordonov S (2012). Immunological Genome C. Gene-expression profiles and transcriptional regulatory pathways that underlie the identity and diversity of mouse tissue macrophages. Nat Immunol.

[12] Murray PJ, Allen JE, Biswas SK, Fisher EA, Gilroy DW, Goerdt S, Gordon S, Hamilton JA, Ivashkiv LB, Lawrence T (2014). Macrophage activation and polarization: nomenclature and experimental guidelines. Immunity.

[13] Serhan CN (2014). Pro-resolving lipid mediators are leads for resolution physiology. Nature.

[14] Serhan CN, Dalli J, Karamnov S, Choi A, Park CK, Xu ZZ, Ji RR, Zhu M, Petasis NA (2012). Macrophage proresolving mediator maresin 1 stimulates tissue regeneration and controls pain. FASEB J.

[15] Zadravec D, Tvrdik P, Guillou H, Haslam R, Kobayashi T, Napier JA, Capecchi MR, Jacobsson A (2011). ELOVL2 controls the level of n-6 28:5 and 30:5 fatty acids in testis, a prerequisite for male fertility and sperm maturation in mice. J Lipid Res.

[16] Chiurchiù V, Lanuti M, De Bardi M, Battistini L, Maccarrone M (2015). The differential characterization of GPR55 receptor in human peripheral blood reveals a distinctive expression in monocytes and NK cells and a proinflammatory role in these innate cells. Int Immunol.

[17] Chiurchiù V, Lanuti M, Catanzaro G, Fezza F, Rapino C, Maccarrone M (2014). Detailed characterization of the endocannabinoid system in human macrophages and foam cells, and anti-inflammatory role of type-2 cannabinoid receptor. Atherosclerosis.

[18] Maskrey BH, Megson IL, Rossi AG, Whitfield DP (2013). Emerging importance of omega-3 fatty acids in the innate immune response: Molecular mechanisms and lipidomic strategies for their analysis. Mol Nutr Food Res.

[19] Yan Y, Jiang W, Spinetti T, Tardivel A, Castillo R, Bourquin C, Guarda G, Tian Z, Tschopp J, Zhou R (2013). Omega-3 fatty acids prevent inflammation and metabolic disorder through inhibition of NLRP3 inflammasome activation. Immunity.

[20] Chang HY, Lee HN, Kim W, Surh YJ (2015). Docosahexaenoic acid induces M2 macrophage polarization through peroxisome proliferator-activated receptor γ activation. Life Sci.

[21] Ali M, Heyob K, Rogers LK (2016). DHA Suppresses Primary Macrophage Inflammatory Responses via Notch 1/ Jagged 1 Signaling. Sci Rep.

[22] Sica A, Erreni M, Allavena P, Porta C (2015). Macrophage polarization in pathology. Cell Mol Life Sci.

[23] McNelis JC, Olefsky (2014). Macrophages, immunity, and metabolic disease. Immunity.

[24] Greenwald RJ, Freeman GJ, Sharpe AH (2005). The B7 family revisited. Annu Rev Immunol.

[25] Mantovani A (2008). From phagocyte diversity and activation to probiotics: back to Metchnikoff. Eur J Immunol.

[26] Martinez FO, Gordon S (2014). The M1 and M2 paradigm of macrophage activation: time for reassessment. F1000Prime Rep.

[27] Dalli J, Ramon S, Norris PC, Colas RA, Serhan CN (2015). Novel proresolving and tissue-regenerative resolvin and protectin sulfido-conjugated pathways. FASEB J.

[28] Chiurchiù V, Leuti A, Dalli J, Jacobsson A, Battistini L, Maccarrone M, Serhan CN (2016). Proresolving lipid mediators resolvin D1, resolvin D2, and maresin 1 are critical in modulating T cell responses. Sci Transl Med.

[29] Rius B, Lopez-Vicario C, Gonzalez-Periz A, Moran-Salvador E, Garcia-Alonso V, Claria J, Titos E (2012). Resolution of inflammation in obesity-induced liver disease. Front Immunol.

[30] Boutens L, Stienstra R (2016). Adipose tissue macrophages: going off track during obesity. Diabetologia.

[31] Yamauchi T, Kamon J, Waki H, Terauchi Y, Kubota N, Hara K, Moy Y, Ide T, Murakami K, Tsuboyama-Kasaoka N (2001). The fat-derived hormone adiponectin reverses insuln resistance associated with both lipoatrophy and obesity. Nat Med.

[32] Al-Hasani H, Joost HG (2005). Nutrition-/diet-induced changes in gene expression in white adipose tissue. Best Pract Res Clin Endocrinol Metab.

[33] Vieira Potter VJ, Strissel KJ, Xie C, Chang E, Bennett G, Defuria J, Obin MS, Greenberg AS (2012). Adipose tissue inflammation and reduced insulin sensitivity in ovariectomized mice occurs in the absence of increased adiposity. Endocrinology.

[34] Martínez-Fernández L, Laiglesia LM, Huerta AE, Martinez JA, Moreno-Aliaga MJ (2015). Omega-3 fatty acids and adipose tissue function in obesity and metabolic syndrome. Prostaglandins Other Lipid Mediat.

